# Active Shiga-Like Toxin Produced by Some *Aeromonas* spp., Isolated in Mexico City

**DOI:** 10.3389/fmicb.2016.01522

**Published:** 2016-09-26

**Authors:** Ingrid Palma-Martínez, Andrea Guerrero-Mandujano, Manuel J. Ruiz-Ruiz, Cecilia Hernández-Cortez, José Molina-López, Virgilio Bocanegra-García, Graciela Castro-Escarpulli

**Affiliations:** ^1^Laboratorio de Bacteriología Médica, Departamento de Microbiología, Escuela Nacional de Ciencias Biológicas, Instituto Politécnico NacionalMexico City, Mexico; ^2^Laboratorio Central de Análisis Clínicos Unidad Médica de Alta Especialidad Hospital de Pediatría “Silvestre Frenk Freund,” Centro Médico Nacional Siglo XXIMexico City, Mexico; ^3^Laboratorio de Bioquímica Microbiana, Departamento de Microbiología, Escuela Nacional de Ciencias Biológicas, Instituto Politécnico NacionalMexico City, Mexico; ^4^Departamento de Salud Pública, Facultad de Medicina, Universidad Nacional Autónoma de MéxicoMexico City, Mexico; ^5^Centro de Biotecnología Genómica, Instituto Politécnico NacionalReynosa, Mexico

**Keywords:** Shiga-like toxin, *Aeromonas* spp., diarrhea, uremic hemolytic syndrome, outer membrane vesicles

## Abstract

Shiga-like toxins (Stx) represent a group of bacterial toxins involved in human and animal diseases. Stx is produced by enterohemorrhagic *Escherichia coli, Shigella dysenteriae* type 1, *Citrobacter freundii*, and *Aeromonas* spp.; Stx is an important cause of bloody diarrhea and hemolytic uremic syndrome (HUS). The aim of this study was to identify the *stx*1*/stx*2 genes in clinical strains and outer membrane vesicles (OMVs) of *Aeromonas* spp., 66 strains were isolated from children who live in Mexico City, and Stx effects were evaluated in Vero cell cultures. The capacity to express active Stx1 and Stx2 toxins was determined in Vero cell cultures and the concentration of Stx was evaluated by 50% lethal dose (LD_50_) assays, observing inhibition of damaged cells by specific monoclonal antibodies. The results obtained in this study support the hypothesis that the *stx* gene is another putative virulence factor of *Aeromonas*, and since this gene can be transferred horizontally through OMVs this genus should be included as a possible causal agents of gastroenteritis and it should be reported as part of standard health surveillance procedures. Furthermore, these results indicate that the *Aeromonas* genus might be a potential causative agent of HUS.

## Introduction

For several years, the significance of *Aeromonas* spp., as a human diarrhea-causing agent was controversial; several studies demonstrated that the pathogenic mechanism of *Aeromonas* is multifactorial because many virulence factors are involved, including the production of cytotoxins (Castro-Escarpulli et al., 2002, 2003). These toxins can cause diarrhea or hemorrhagic colitis, and may play a major role in the hemolytic-uremic syndrome (HUS) and TTP development (Bogdanović et al., 1991; Fang et al., 1999; Monforte-Cirac et al., 2010).

The cytotoxins implicated in these diseases include Shiga toxin and the closely related Stx. Stx variants are expressed in *Shigella, Enterobacter, Citrobacter, Acinetobacter, Campylobacter*, and *Hamiltonella* bacterial species (Mauro and Koudelka, 2011). Alperi and Figueras (2010) described the presence of Stx1 and Stx2 in clinical isolates of *Aeromonas* spp., associated with gastroenteritis, hemorrhagic colitis, and HUS. Genes encoding these toxins are located in different lambdoid bacteriophages that lysogenize this strain. In addition, the genus *Aeromonas* has a zero-secretion system named OMVs. OMVs could be a means by which some proteins, RNA, periplasmic space components and other components associated with virulence, may be transferred horizontally to other genera; therefore, it is believed that OMVs play an important role in pathogenicity (Guerrero-Mandujano et al., 2015a,b).

For this reason, the aim of this study was to evaluate the damage caused by the production of Stx by strains isolated from Mexico City children in Vero cell cultures.

## Materials and Methods

### Strains

This study included 66 clinical isolates from the INP, 54 obtained from intestinal and 12 from extra-intestinal infections. Strains were isolated from specimens obtained for routine testing at the mentioned hospital; therefore, no informed consent was required from parents or legal guardians of children. All strains were genetically identified by 16S rDNA-RFLP (Hernández-Cortez et al., 2011). The typed strain for *Escherichia coli* O157:H7 CECT 4076 was used as the positive control and *E. coli* K12 strain (5512 ENCB) from the collection of the Medical Bacteriology Laboratory *(Escuela Nacional de Ciencias Biologicas, IPN*) was used as the negative control for toxin production. The strains were maintained for short periods at room temperature on blood agar based slants; for longer storage, they were either frozen at -70°C in 20% (w/v) glycerol-Todd-Hewitt broth (Oxoid, Mexico) or lyophilized in 7.5% horse glucose serum.

### DNA Extraction

All the cultures were grown on tryptic soy agar at 30°C for 18 h. The genomic DNA of each strain was obtained through InstaGene Matrix (BioRad^®^, Mexico) according to the instructions provided by the manufacturer. DNA purity and quantity were determined by using the Ampli Quant AQ-07 spectrophotometer. DNA was stored at -20°C until use.

### *stx1* and *stx2* PCR Amplifications

The presence of *stx1 and stx2* from DNA of OMVs and genomic DNA was detected by single PCR reactions using primers STX1F/STX1R and STXF/STXR with a 144 and 217 bp product, respectively, these primers were designed based on the sequence of subunit A. The primers, the reaction, and amplification conditions were processed as previously described by Hernández-Cortez et al. (2013), with the positive (*E. coli* O157:H7) and negative (*E. coli* K12) controls.

### DNA Sequencing

Polymerase chain reaction products were purified using a PureLink Quick Gel Extraction Kit (Invitrogen^®^, Mexico) according to manufacturer’s instructions. The products were directly sequenced on an ABI-PRISM 310 Genetic Analyzer (Applied Biosystems, Foster City, CA, USA) using the forward and reverse primers used for PCR, according to manufacturer’s instructions. Sequencing was performed at the *Instituto de Biología, UNAM* (Mexico). Sequence analysis was performed with the Basic Local Alignment Search Tool (BLAST) provided by the National Center for Biotechnology Information (NCBI).

### Microplate Vero Cells Preparations

This procedure was performed in 96-well microplates with Vero (ATCC CCL81) cell monolayer with 80% confluence, adding minimal essential medium (MEM; Invitro^®^, Mexico) supplemented with 10% v/v fetal bovine serum (FBS; Invitro^®^, Mexico).

The cell suspension was homogenized and adjusted to 10^5^–10^6^ cells/mL using a Neubauer chamber. After adjusting, the suspension was deposited in 200-μL well. The microplates were incubated at 37°C under 5% CO_2_ for 24 h (CO_2_ Incubator, VWR Scientific, USA) (Giono-Cerezo et al., 1994).

### Cell-Free Bacterial Preparations

Five colonies from each blood agar plate were inoculated into 3 mL of Craig medium (0.4% yeast extract, 3% casamino acids, 0.05% K_2_HPO_4_). These were incubated for 24 h at 37°C and the optical density of the bacterial culture used was 0.25 at 600 nm. Cell-free preparations were made by centrifuging the cultures at 14,000 *g* for 10 min at 4°C, followed by filtration of the supernatant through a membrane filter (pore size 0.45 pm, Sartorius Minisart NML). Cell-free supernatants were stored at -20°C. A total of 66 cell-free bacterial preparations were obtained in this way; the positive control (*E. coli* O157:H7) and the negative control (*E. coli* K12) were obtained also in the same way (Giono-Cerezo et al., 1994).

### Cytotoxic Assay and LD_50_ Determination

The cell-free filtrate (20 μL) was inoculated into wells containing cells and the respective growth medium without antibiotics. Inoculated cells were incubated for 96 h at 37°C with 5% CO_2_ and observed every 24 h on the inverted microscope. The cytotoxic effect was expected to appear as rounding and shrinkage of cells with thick granulation and, finally, progressive and irreversible destruction of the monolayer. All tests were performed in duplicate; viability controls with MEM and Craig medium were performed also (Giono-Cerezo et al., 1994).

The LD_50_ was determined in *Aeromonas* strains detected as positive for causing cytotoxic damage in Vero cells. A standard 96-well microplate with Vero cells was prepared as indicated in 2.6, but the medium was changed to 100 μL of MEM with 1% BFS. This preparation was exposed to 100 μL of cell -free bacterial preparation, and serial dilutions were done on the whole row of plates. After 24 h of incubation at 37°C with 5% CO_2_, the LD_50_ was determined, under the inverted microscope. LD_50_ was assigned to the well in which 50% of Vero cells were damaged and 50% un-damaged (Marques et al., 1986).

### Blockade and Toxin Neutralization Assay

The toxin neutralization assay was performed in the strains that induced cytotoxic damage in Vero cells because this damage is indicative of Stx. For this test, a microplate was prepared with Vero cell grown to a confluence of 90–100% with the LD_50_ of each cell-free bacterial preparation. To perform the neutralization assay to show cell damage produced as a consequence of Stx action, two monoclonal antibodies (*Universidad Nacional Autónoma de México*) obtained from *E. coli* O157:H7 were used.

For each Ab a Bradford protein quantitation was done according to manufacturer’s instructions (Biorad), with a result of 78.2 and 74.8 μg/μL, respectively, of anti-Stx1 and anti-Stx2 A. The concentrated Ab was worked and a double dilution of the Ab. Then, 10 μL of the Ab (anti-STX1 or anti-STX2) was incubated with 190 μL of the cell-free supernatant at a concentration of LD_50_ for 1 h at 37°C. Following this, 200 μL of the latter was inoculated into Vero cells at 90% of confluence and incubated for 24 h at 37°C with 5% CO_2_ (Marques et al., 1986).

### OMVs Procurement and DNA Extraction from OMVs

Outer membrane vesicles were obtained from *Aeromonas hydrophila* F-0050. The protocol was performed as previously described by Guerrero-Mandujano et al. (2015a).

The OMVs’ DNA was obtained through InstaGene Matrix (BioRad^®^, Mexico) according to the instructions provided by the manufacturer. Then, the OMVs’ DNA was purified with the DNA extraction phenol-chloroform technique (Guerrero-Mandujano et al., 2015b).

## Results

### *stx1* and *stx2* Gene PCR Amplifications

Polymerase chain reaction screening of 66 clinical *Aeromonas* strains showed that 22/66 (33.3%) strains contained the *stx1* gene, 42/66 (63.6%) strains contained both genes, no strains contained only the *stx2* gene, and 2/66 (3%) strains were negative for both genes.

### DNA Sequencing

BLASTn analysis showed a 79 to 99% similarity and an expected value of 3e-16/2e-97 between the *stx-1/stx-2* genes of *E. coli* O157:H7 and the amplicon from *Aeromonas* spp., strains.

### Cytotoxic Assay and LD_50_ Determination

**FIGURE 1:**
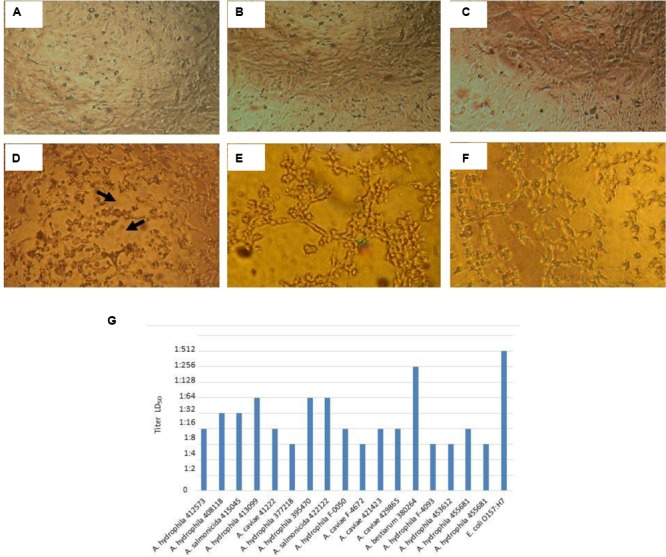
**Cytotoxicity assay and LD_50_ determination performed in Vero cell cultures. (A)** Vero cell culture with Minimal Essential Medium (Viability test). **(B)** Vero cell culture with Craig Medium (Viability test). **(C)** Negative control with *E. coli* K12. **(D)** Positive control with *E. coli* O157: H7, Cytotoxic damage is shown (arrows) by destruction of the cell monolayer and “old lace” effect. **(E)** Cytotoxic damage of *A. hydrophila* F-0050 strain. **(F)** Cytotoxic damage of *A. caviae* 421423 strain. **(G)** Graphical representation of the LD_50_ titer of all strains.

The cytotoxicity test performed in Vero cell cultures showed that 17/66 (25.7%) cell-free bacterial preparations caused cytotoxic damage, suggesting production of an active Stx (**Figure 1**), revealed by the characteristic damage caused by Stx.

The results for determining the LD_50_ titer were as follows: 6/17 (35.2%) cell-free bacterial preparations presented a titer of 1:16; 5/17 (29.4%) cell-free bacterial preparations depicted a titer of 1:8; 3/17 (17.6%) cell-free bacterial preparations had a titer of 1:64; 2/17 (11.7%) cell-free bacterial preparations with a titer of 1:32; and 1/17 (5.8%) cell-free bacterial preparations with a titer of 1:256 (**Figure 1G**).

### Toxin Neutralization

**FIGURE 2:**
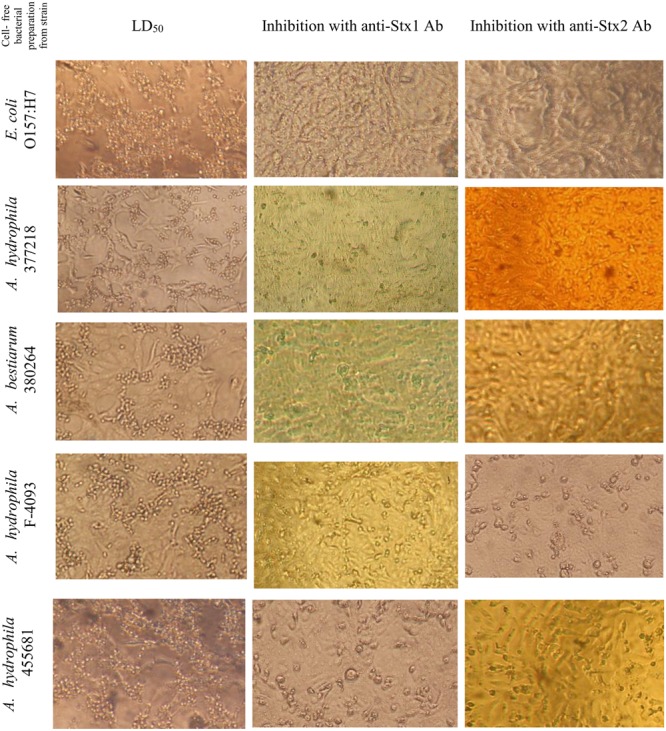
**Inhibition of cytotoxic effect induced in Vero cells culture by cell-free bacterial preparations of *Aeromonas* spp. strains bearing the *stx1* or *stx2* gene**.

**FIGURE 3:**
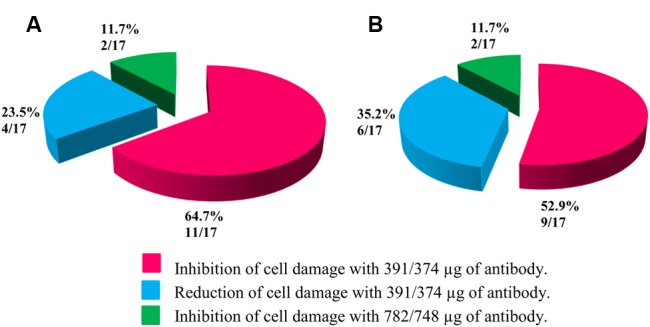
**Percentage of strains that showed inhibition or reduction of cell damage. (A)** Neutralization with the anti-Stx1 Ab. **(B)** Neutralization with the anti-Stx2 Ab.

The application of anti-STX1 Ab in 11/17 (64.7%) strains the damage to cells in the supernatant was completely inhibited at 39.1 μg/μL Ab concentration; in 4/17 (23.5%) cell-free bacterial preparations, reduced cell damage was observed at the same concentration; and in 2/17 (11.7%) cell-free bacterial preparations, cell damage was completely inhibited at a concentration 78.2 μg/μL. Using the Ab anti-STX2 showed that in 9/17 (52.9%) cell-free bacterial preparations, cell damage was totally inhibited at 37.4 μg/μL, and 6/17 (35.2%) cell-free bacterial preparations showed reduced cell damage at the same concentration; and in 2/17 (11.7%) cell-free bacterial preparations, cell damage was completely inhibited with an Ab concentration of 74.8 μg/μL (**Figures 2** and **3**).

### *stx1* Gene PCR Amplifications from OMVs

**FIGURE 4:**
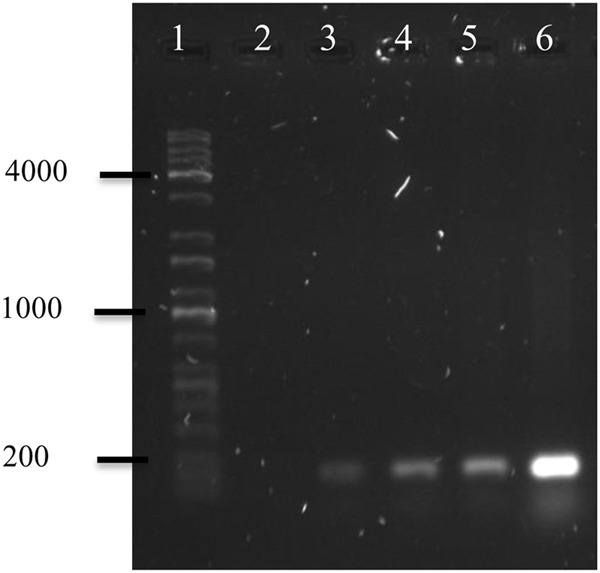
**Electropherogram of *stx1* gene amplification from OMVs’ DNA.** (1) Kapa Universal Ladder, (2) Negative control, (3) OMVs of *A. hydrophila* F-0050, (4) *A. hydrophila* F-0050, (5) OMVs of *E. coli* O157:H7, and (6) *E. coli* O157:H7.

Polymerase chain reaction of OMVs’ DNA of *A. hydrophila* F-0050 revealed the OMVs contained the gen *stx1*; hence, OMVs might be a potential vehicle for horizontal virulence genes transfer (**Figure 4**).

## Discussion

The genus *Aeromonas*, as a producer of Stx, could be an emergent pathogen that causes diarrhea mainly in pediatric patients (Hernández-Cortez et al., 2011; Figueras and Baez-Hidalgo, 2014). Nevertheless, this genus has become more relevant in the medical area, as it is a pathogen causing HUS (San Joaquín and Pickett, 1988; Bogdanović et al., 1991; Robson et al., 1992; Fang et al., 1999; Monforte-Cirac et al., 2010).

In this study, we found, by PCR amplification of the *stx1* and *stx2* genes, that 22/66 (33.3%) of the aeromonad strains contained the *stx1 gene*, 42/66 (63.6%) strains contained both genes, and there were no strains containing *stx2* alone. This can lead us to suggest that this gene is widely distributed in the *Aeromonas* strains isolated from pediatric patients and that these patients can be at risk for developing HUS.

There is only one previous report in which the *stx* genes were studied in *Aeromonas* strains isolated from different hospitals in Spain; the results obtained were that 19/80 (23.7%) strains had the *stx1* gene and only 1/80 (1.25%) strain was positive for both genes (Alperi and Figueras, 2010). These results convey that there is a greater distribution of the gene in Mexican strains; however, we should take into account that, in Spain, the *Aeromonas* genus is a known gastrointestinal pathogen isolated routinely in patients with a gastrointestinal profile, therefore, an adequate treatment is given to confront this genus.

In Mexico, this genus is not included in the list of pathogens causing diarrhea and it is not isolated routinely; as a result, it is difficult to give any treatment and good quality of life to patients; moreover, there are no statistics about its prevalence. In addition, the genus becomes more relevant due to the socioeconomic conditions of the Mexican population, which include water shortage; this facilitates its transmission and increases the possibility of HUS becoming a public health problem.

Sequencing of the amplicons was carried out and a Blast search was performed, which showed 79–99% similarity and an expectancy value of 3e-16/2e-97 between genes *stx1/stx2* as compared to *E. coli* O157:H7.

The LD_50_ was determined to evaluate the cytotoxic damage in Vero cell culture; with this parameter and based on Marques et al. (1986), the strains can be grouped in low (2 × 10^1^ to 6 × 10^2^), moderate (10^3^–10^4^), and high (10^5^–10^8^) production (**Figure 1G**). Marques et al. (1986) carried out a study in which they made a similar assay to that of the present study, but with different strains of STEC, they obtained that 262/400 (63%) strains were grouped as low Stx producers, and 48% produced cytotoxic damage; nevertheless, in 40% of these strains the cytotoxic damage was inhibited with anti-Stx antibodies.

In the present study, 25.7% of bacteria-free preparations caused cytotoxic damage characteristic of Stx in Vero cells, suggesting production of active Stx, all *Aeromonas* strains were grouped as low producers of Stx; however, in 50–70% of the cell-free supernatants, the cellular damage was inhibited, and in 20–30% of cell-free supernatants the cellular damage was reduced (**Figure 3**). The cell-free supernatants in which cellular damage was reduced this could have been because of two variants in the toxin present in the supernatant, consequently, as they are different immunologically, the Stx could not be totally inhibited and the cellular damage remained (Marques et al., 1986).

Two previous studies (Haque et al., 1996; Alperi and Figueras, 2010) obtained 10.2% and 10.53% of Stx1-producing *Aeromonas* strains, respectively. The reason for a higher percentage of *Aeromonas* strains producing active Stx could be due to the high availability of the Stx bacteriophage inside the culture as some STEC strains carriers of the Stx bacteriophage have been isolated from urban wastewater from treatment plants, wastewater from slaughterhouses and cattle stools (García-Aljaro et al., 2004, 2009). Similarly transduction studies have been carried out *in vivo* with Stx phages from *E. coli*, which can infect intestinal microbiota bacteria, giving the toxigenic characteristic to strains that were not infected before (Acheson et al., 1998; Schmidt et al., 1999; Gamage et al., 2003). The same could happen with the genus *Aeromonas* when inducing a gastrointestinal profile, followed by the fact that the genus *Aeromonas* has the ability to capture and integrate virulence factors in its genome, and one of these is the Stx-encoding gene.

The rest of the strains caused cytotonic damage in the cell, possibly suggesting production of different toxins other than Stx, since *Aeromonas* is a genus capable of producing two cytotonic enterotoxins, thermostable (AST) and thermolabile (ALT) (Figueras and Baez-Hidalgo, 2014), that can cause cytotonic damage in Vero cells; another possibility is that the *Aeromonas* strains have more than one bacteriophage inserted in their genome, as it has been demonstrated that STEC strains with double lysogeny are able to regulate bacteriophages to inhibit the capacity of lytic cycle induction, which results in a decrease of Stx production but without losing the gene (García-Aljaro et al., 2004; Imamovic and Muniesa, 2011). Therefore, it is necessary to establish how many bacteriophages do *Aeromonas* strains contain, including in the present study, to be able to correlate the presence of Stx bacteriophages and the amount produced of Stx.

On the other hand, the presence of Stx bacteriophages in inducible *Aeromonas* represents a horizontal transfer mechanism of the *stx* gene, but it is not the only one. In more recent studies, it has been determined that *Aeromonas* are capable of producing OMVs (Guerrero-Mandujano et al., 2015a) and these OMVs are capable of transporting integrated DNA (Guerrero-Mandujano et al., 2015b). In the present study, we amplified the *stx1* gene from the DNA extracted from the OMVs of one *A. hydrophila* strain of clinical origin, which induced cytotoxic damage in Vero cells, and this damage was inhibited with anti-Stx antibodies. This indicates that the Stx bacteriophage is the transport mechanism of the *stx* gene; furthermore, the OMVs and the recently called Transport System Type 0 are capable of spreading the *stx* gene interspecies or intraspecies. Nevertheless, it is required to confirm if the gene contained in the OMVs is complete. The results obtained in this study support the hypothesis that the *stx* gene is another putative virulence factor of *Aeromonas* that might be transferred through OMVs. It could represent another mechanism for horizontal transport of the *stx* gene to other *Aeromonas* strains and/or other bacterial genera.

For this reason, this genus should be included as possible causative agent of gastroenteritis when trying to identify causal agents and should be reported as part of standard health surveillance procedures.

## Author Contributions

IP-M and AG-M performed the experiments and MR-R performed the bioinformatics analyses and drafted the manuscript; CH-C designed the primer; VB-G, and JM-L critically commented and revised the manuscript; GC-E conceived the study, participated in its design and coordination, assessed the data and drafted the manuscript. All authors read and approved the final manuscript. We would like to thank Sofia Marteli Mulia for kindly correcting the style of the manuscript.

## Conflict of Interest Statement

The authors declare that the research was conducted in the absence of any commercial or financial relationships that could be construed as a potential conflict of interest.

## Abbreviations

Ab: Antibody
HUS: Hemolytic uremic syndrome
INP: Instituto Nacional de Pediatria Mexico
LD_50_: Median lethal dose
OMVs: Outer membrane vesicles
PCR: Polymerase chain reaction
STEC: Shiga toxin-producing *E. coli*
Stx: Shiga-like toxin
TTP: Thrombotic thrombocytopenic purpura
